# Smoking-attributable low-back pain disability in China versus high income countries among adults aged 20–54 years, 1990–2023: A secondary dataset analysis of GBD 2023

**DOI:** 10.18332/tid/219036

**Published:** 2026-06-12

**Authors:** Yushan Zhu, Zhiwei Li, Kainan Li, Rushuo Wei, Tiangang Zhou, Sen Yang, Mingdong Yu, Bingwu Wang

**Affiliations:** 1 Department of Spinal Surgery, Weifang People’s Hospital, Shandong Second Medical University, Weifang, Shandong, China; 2 Digital Spine and Minimally Invasive Research Institute, Shandong Second Medical University, Weifang, Shandong, China

**Keywords:** low-back pain, smoking, population attributable fraction, years lived with disability, Global Burden of Disease

## Abstract

**INTRODUCTION:**

Low-back pain (LBP) is a leading cause of disability. This study compared temporal trends in working-age LBP burden and smoking-attributable disability between China and the GBD aggregate ‘High-income countries’ from 1990 to 2023, with high body-mass index (BMI) and occupational ergonomic exposures as contextual risk factors.

**METHODS:**

This secondary analysis used Global Burden of Disease (GBD) 2023 estimates for adults aged 20–54 years in China and high-income countries. Annual incidence, prevalence and years lived with disability (YLD) were extracted as counts and age-standardized rates per 100000 population (95% uncertainty intervals [UI]). Trend magnitude was summarized using joinpoint regression (average annual percent change [AAPC], 95% confidence interval [CI]) applied to annual point estimates. Risk-attributable YLD rates and population attributable fractions (PAFs) for smoking, high BMI and occupational ergonomic exposures were obtained from the GBD comparative risk assessment.

**RESULTS:**

In 2023, age-standardized incidence and YLD rates were higher in high-income countries (5115.98 per 100000, 95% UI: 4396.74–5819.09; 1396.92 per 100000, 95% UI: 993.01–1842.94) than in China (2497.71 per 100000, 95% UI: 2086.63–2942.72; 656.08 per 100000, 95% UI: 448.25–884.77). Rates declined in both settings, faster in China (incidence AAPC= -0.46%, 95% CI: -0.55 – -0.38; YLD AAPC -0.45%, 95% CI: -0.54 – -0.36) than in high-income countries (incidence AAPC= -0.12%, 95% CI: -0.13 – -0.11; YLD AAPC= -0.13%, 95% CI: -0.16 – -0.10). Smoking-attributable PAF decreased in high-income countries from 24.05% (95% UI: 16.04–32.23) to 19.60% (95% UI: 12.70–27.14) but rose slightly in China from 16.61% (95% UI: 11.33–21.74) to 18.27% (95% UI: 12.85–23.88).

**CONCLUSIONS:**

Working-age LBP rates declined in both settings, with larger reductions in China. Smoking-attributable burden also declined, but smoking’s proportional contribution decreased in high-income countries and rose slightly in China. These model-based patterns may inform population-level prevention priorities alongside rising BMI-related and work-related risks.

## INTRODUCTION

Low-back pain (LBP) is a major contributor to non-fatal health loss globally and remains a leading cause of years lived with disability (YLD) across countries and age groups^1^. Its population burden varies by time and setting, reflecting differences in occupational structures, health-system capacity, and behavioral and metabolic risk profiles^2^. Working-age adults (aged 20–54 years) represent the core labor force and account for a substantial share of disability, making this group a priority for prevention and policy planning^3,4^. The age range of 20–54 years was selected to focus on prime working-age disability and to enable consistent comparisons across settings using standard adult age strata, rather than the broader 15–64 years definition.

Smoking is a modifiable behavioral exposure that has been consistently associated with LBP and related degenerative spinal processes in epidemiological and experimental literature^5,6^. Accordingly, tobacco control may have relevance beyond cardiopulmonary outcomes by contributing to musculoskeletal disability prevention^7^. However, tobacco control is typically evaluated using cardiometabolic and respiratory outcomes; whether smoking-attributable LBP disability has followed similar trajectories across major settings – particularly China versus high-income countries – remains poorly characterized ^8^.

Using Global Burden of Disease (GBD) 2023 estimates, this study aimed to compare temporal trends in LBP burden and the smoking-attributable contribution between China and the GBD aggregate ‘High-income countries’ from 1990 to 2023 among adults aged 20–54 years. Incidence, prevalence and years lived with disability (YLD) were examined, long-term and recent trends were summarized using joinpoint regression^9^, and population-attributable fractions (PAFs) for smoking, high BMI and occupational ergonomic exposures were assessed within the GBD comparative risk assessment framework^10^. Smoking-attributable disability was treated as the primary tobacco-relevant signal, while metabolic and occupational risks were retained to contextualize shifts in risk composition and avoid single-factor inference.

## METHODS

### Data sources and study design

This study is a secondary analysis of publicly available Global Burden of Disease (GBD) 2023 estimates produced by the Institute for Health Metrics and Evaluation (IHME), which provide modeled cause- and risk-specific burden by age, sex, location and year (1990–2023)^10^. Eligible records comprised annual estimates for the cause ‘low-back pain’ for China and the GBD aggregate ‘High-income countries’. The primary analytic population was adults aged 20–54 years (seven 5-year age groups: 20–24 to 50–54 years), with all-age estimates used to contextualize working-age findings. Because analyses used de-identified, aggregated secondary data, ethics approval was not required. Reporting followed the Strengthening the Reporting of Observational Studies in Epidemiology (STROBE) guidelines^11^.

Using the GBD Results Tool, incidence, prevalence and years lived with disability (YLD) were retrieved as counts and age-standardized rates per 100000 population with 95% uncertainty intervals (UI) taken directly from GBD outputs^6^. Query settings were: location (China; and ‘High-income countries’ as an aggregate location in GBD), years (1990–2023), sex (both, males, females), age (20–24 to 50–54 years for primary analyses; all ages for contextual analyses), measures (incidence, prevalence, YLD), and metrics (number and rate). Age-standardized rates were those provided by GBD and standardized to the GBD world standard population (i.e. not recalculated in this study).

### Case definition

The GBD case definition of LBP was adopted and operationalized as lumbar-region pain (between the lower rib margin and the gluteal folds) of at least 1-day duration, regardless of whether leg pain was present^7^.

### Measures

LBP burden was characterized using GBD-derived incidence, prevalence and YLD, reported as counts and age-standardized rates per 100000 population. Uncertainty was summarized using 95% uncertainty intervals (UI) provided by GBD^6^.

### Risk-factor attribution

Comparative risk assessment outputs were extracted for smoking, high body-mass index (BMI) and occupational ergonomic exposures, including risk-attributable YLD rates (per 100000) and population attributable fractions (PAFs)^6,12^. In the GBD framework, the PAF represents the proportion of YLD that would be reduced if population exposure were shifted to the theoretical minimum risk exposure level for the corresponding risk factor^13^. Because attribution estimates are model-based, 95% UI may be wide; for some attributable metrics, lower bounds can fall below zero, reflecting statistical uncertainty around counterfactual attribution and values compatible with 0 rather than evidence of a protective effect.

High-income subregion heterogeneity The GBD ‘High-income countries’ location is an aggregate that differs from World Bank income groupings and comprises five subregions: Australasia, High-income Asia Pacific, High-income North America, Western Europe, and Southern Latin America. Sensitivity analyses were conducted across these five subregions to assess heterogeneity within the aggregate. For parsimony, subregional analyses were restricted to adults aged 20–54 years with both sexes combined. Annual YLD counts and age-standardized YLD rates (1990–2023) were summarized using 1990 and 2023 levels, relative change, and joinpoint trend estimates; subregional risk-attribution patterns (PAFs) are presented descriptively in the Supplementary file.

### Statistical analysis

Joinpoint regression (Joinpoint Regression Program, version 5.1.0; National Cancer Institute) was used to estimate annual percent change (APC) and average annual percent change (AAPC) for 1990–2023 using log-linear models fitted to annual age-standardized rates^9,14,15^. Up to three joinpoints were allowed, and model selection followed the software’s permutation-test procedure with two-sided α=0.05. Joinpoint analyses were performed on annual point estimates; thus, APC/AAPC uncertainty does not propagate the full 95% UI of GBD modelled estimates. Data processing and figure preparation were conducted in R (version 4.5.1)^16^.

## RESULTS

LBP incidence, prevalence and YLD among adults aged 20–54 years in China and high-income countries were assessed from 1990 to 2023. Temporal trends were summarized using joinpoint-derived APC and AAPC. High-income subregional estimates were additionally examined to characterize heterogeneity within the high-income aggregate comparator.

### Overall burden in 2023

Working-age LBP burden differed substantially between China and high-income countries (Table 1). In 2023, age-standardized incidence and prevalence rates were higher in high-income countries than in China and the same pattern was observed for YLD rates. The total incidence, prevalence and YLD among individuals aged 20–54 years in high-income countries were 25526161 (95% UI: 21937511–29034325), 61028235 (95% UI: 52589270–69368390) and 6969939 (95% UI: 4954643–9195342), respectively. In contrast, China’s total incidence was 17071120 (95% UI: 14261489–20112623), the prevalence was 38787687 (95% UI: 31973704–45698250), and the YLD were 4484116 (95% UI: 3063668–6047164).

**Table 1:** Burden of low-back pain among adults aged 20–54 years in China and GBD high-income countries, 1990 and 2023, secondary analysis of GBD 2023 estimates

*Location*	*Measure*	*1990 Cases (95% UI)*	*1990 Rate/100k (95% UI)*	*2023 Cases (95% UI)*	*2023 Rate/100k (95% UI)*	*Cases change % (95% UI)*	*AAPC (95% CI)*
**China**	Incidence	17135307 (14478610–19993011)	2906.79 (2456.11–3391.56)	17071120 (14261489–20112623)	2497.71 (2086.63–2942.72)	-0.37 (-7.51–7.89)	-0.46* (-0.55 – -0.38)
	Prevalence	38888529 (32518027– 45908176)	6596.95 (5516.27–7787.74)	38787687 (31973704–45698250)	5675.11 (4678.14–6686.20)	-0.26 (-7.61 – -7.29)	-0.46* (-0.55 – -0.37)
	YLD	4482112 (3038328–6068718)	760.33 (515.41–1029.48)	4484116 (3063668–6047164)	656.08 (448.25–884.77)	0.04 (-7.55–8.23)	-0.45* (-0.54 – -0.36)
**Highincome**	Incidence	24045155 (20513872–27765930)	5335.47 (4551.90–6161.08)	25526161 (21937511–29034325)	5115.98 (4396.74–5819.09)	6.16 (3.05–9.84)	-0.12* (-0.13 – -0.11)
	Prevalence	57559683 (49020896–66253952)	12772.13 (10877.43–14701.33)	61028235 (52589270–69368390)	12231.34 (10540.00–13902.89)	6.16 (3.05–9.84)	-0.12* (-0.16 – -0.09)
	YLD	6589592 (4563598–8867155)	1462.19 (1012.63–1967.57)	6969939 (4954643–9195342)	1396.92 (993.01–1842.94)	5.77 (2.61–9.70)	-0.13* (-0.16 – -0.10)

Rates are age-standardized per 100000 population with 95% UI. Cases change (%) = [(2023-1990)/1990]×100. AAPC (1990–2023) was estimated using joinpoint regression on annual point estimates and is reported with 95% CI.

* Statistically significant at p<0.05. AAPC: average annual percent change. CI: confidence interval. UI: uncertainty interval. YLD: years lived with disability.

### Long-term trends

**Figure 1:**
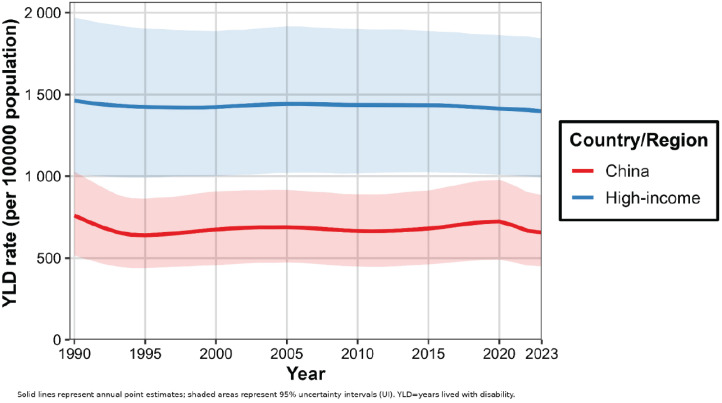
Age-standardised low back pain YLD rate (per 100000) among adults aged 20–54 years in China and the GBD aggregate 'High-income countries', 1990–2023 (GBD 2023 estimates)

Across 1990–2023, age-standardized incidence, prevalence and YLD rates declined in both settings, with consistently larger long-term reductions in China than in high-income countries (Figure 1 and Table 1; and Supplementary file Figure S1). In high-income countries, the age-standardized incidence rate decreased from 5335.47 per 100000 (95% UI: 4551.90–6161.08) in 1990 to 5115.98 (95% UI: 4396.74–5819.09) in 2023, with an AAPC of -0.12% (95% CI: -0.13 – -0.11; p<0.001). The age-standardized prevalence rate decreased from 12772.13 (95% UI: 10877.43–14701.33) to 12231.34 (95% UI: 10540.00–13902.89), AAPC= -0.12% (95% CI: -0.16 – -0.09; p<0.001). The age-standardized YLD rate decreased from 1462.19 (95% UI: 1012.63–1967.57) to 1396.92 (95% UI: 993.01–1842.94), AAPC= -0.13% (95% CI: -0.16 – -0.10; p< 0.001).

In China, the age-standardized incidence rate decreased from 2906.79 (95% UI: 2456.11–3391.56) to 2497.71 (95% UI: 2086.63–2942.72), with an AAPC of -0.46% (95% CI: -0.55 – -0.38; p<0.001). The age-standardized prevalence rate decreased from 6596.95 (95% UI: 5516.27–7787.74) to 5675.11 (95% UI: 4678.14–6686.20), AAPC= -0.46% (95% CI: -0.55 – -0.37; p<0.001). The age-standardized YLD rate decreased from 760.33 (95% UI: 515.41–1029.48) to 656.08 (95% UI: 448.25–884.77), AAPC= -0.45% (95% CI: -0.54 – -0.36; p<0.001) (Figure 1 and Table 1; and Supplementary file Figure S1).

### Recent trends

Joinpoint regression indicated accelerated declines in recent periods in both settings (Supplementary file Figure S2). In high-income countries, the APC for incidence during 2021–2023 was -0.50% (95% CI: -0.58 – -0.41; p<0.001), for prevalence during 2015–2023 it was -0.27% (95% CI: -0.29 – -0.24; p<0.001), and for YLD during 2016–2023 it was -0.34% (95% CI: -0.37 – -0.32; p<0.001). In China, the APC for incidence from 2020 to 2023 was -3.47% (95% CI: -4.02 – -2.91; p<0.001), for prevalence it was -3.50% (95% CI: -4.10 – -2.92; p<0.001), and for YLD it was -3.52% (95% CI: -4.11 – -2.92; p<0.001).

### Sex-specific patterns

Sex-stratified analyses showed distinct patterns between high-income countries and China, in both long-term trends (AAPC) and recent joinpoint-defined segments (APC). In high-income countries, burdens declined in both sexes from 1990 to 2023, with AAPCs for males of -0.14% (95% CI: -0.16 – -0.12; p<0.001) for incidence, -0.13% (95% CI: -0.16 – -0.11; p<0.001) for prevalence, and -0.14% (95% CI: -0.16 – -0.12; p<0.001) for the YLD rate; corresponding female AAPCs were -0.10% (95% CI: -0.12 – -0.09; p<0.001), -0.11% (95% CI: -0.14 – -0.08; p<0.001), and -0.12% (95% CI: -0.16 – -0.09; p<0.001) (Supplementary file Figure S3). Recent joinpoint segments suggested further acceleration in both sexes (Supplementary file Figure S4).

In China, long-term declines were larger among females. Male AAPCs were -0.26% (95% CI: -0.34 – -0.18; p<0.001) for incidence, -0.16% (95% CI: -0.25 – -0.08; p<0.001) for prevalence, and -0.16% (95% CI: -0.25 – -0.07; p<0.001) for the YLD rate; female AAPCs were -0.63% (95% CI: -0.72 – -0.54; p<0.001), -0.70% (95% CI: -0.80 – -0.60; p<0.001), and -0.69% (95% CI: -0.78 – -0.59; p<0.001), respectively (Supplementary file Figure S3). During 2020–2023, female APCs were -4.06% (95% CI: -4.67 – -3.43; p<0.001) for incidence, -4.05% (95% CI: -4.69 – -3.41; p<0.001) for prevalence, and -4.09% (95% CI: -4.72 – -3.46; p<0.001) for the YLD rate; male APCs were -2.77% (95% CI: -3.27 – -2.26; p<0.001), -2.87% (95% CI: -3.41 – -2.34; p<0.001), and -2.89% (95% CI: -3.44 – -2.34; p<0.001), respectively (Supplementary file Figure S5).

### Age-specific trends

**Figure 2:**
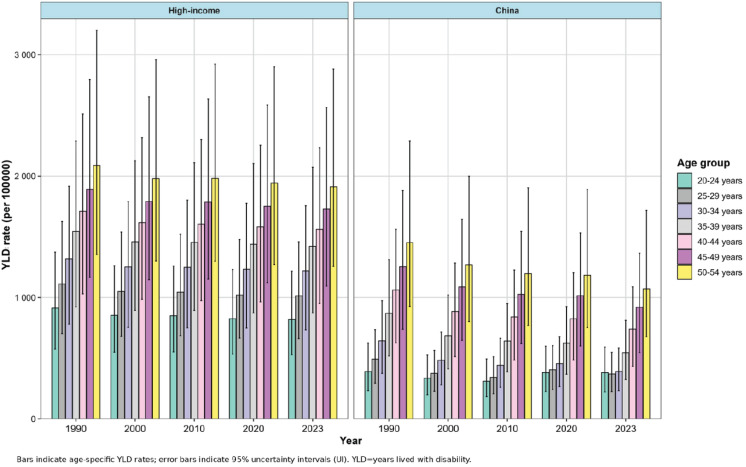
Age-specific low back pain YLD rates (per 100000) across adults aged 20–54 years in China and the GBD aggregate 'High-income countries' in 1990, 2000, 2010, 2020, and 2023 (GBD 2023 estimates)

Age-specific patterns of LBP-related YLD rates differed between high-income countries and China (Figure 2). In high-income countries, YLD rates rose with age across working adulthood and the age gradient changed little over time. In 2023, the YLD rate in the age group of 50–54 years was 1911.95 per 100000 (95% UI: 1255.16–2881.75), 2.3-fold that of the age group of 20–24 years group (818.24 per 100000; 95% UI: 527.80–1218.48). In China, the age profile was less smooth but the age group of 50–54 years consistently showed the highest working-age YLD rate. In 2023, the YLD rate in the age group of 50–54 years group was 1071.99 per 100000 (95% UI: 676.71–1717.80), 2.8-fold that of the age group of 20–24 years (380.71 per 100000; 95% UI: 221.96–592.74).

### All-age comparison

To contextualize working-age findings, all-age estimates were summarized (Supplementary file Figure S6 and Table S1). In 2023, the all-age age-standardized incidence rate was 4166.31 per 100000 (95% UI: 3749.45–4563.83) in high-income countries and 2164.80 (95% UI: 1926.65–2383.57) in China, with similar disparities for prevalence (9844.18; 95% UI: 8911.50–10790.61 vs 4929.78; 95% UI: 4304.39–5494.10) and YLD rates (1095.83; 95% UI: 775.15–1467.99 vs 551.92; 95% UI: 388.90–750.82). Across 1990–2023, all-age age-standardized YLD rates declined in both settings, with a steeper reduction in China (AAPC= -0.89%; 95% CI: -0.95 – -0.83; p<0.001) than in high-income countries (AAPC= -0.22%; 95% CI: -0.25 – -0.20; p<0.001), while all-age YLD counts increased by 37.55% (95% UI: 30.39–45.05) in China and by 31.32% (95% UI: 27.54–35.53) in high-income countries. Adults aged 20–54 years accounted for 42.16% (95% UI: 22.16–62.16) of all-age LBP YLD in China and 43.11% (95% UI: 24.20–62.02) in high-income countries. Age-specific YLD rates rose steeply with age, peaking at 75–79 years in high-income countries and 85–89 years in China; rates were consistently higher in high-income countries across age strata, and uncertainty widened at older ages (Supplementary file Figure S7).

### Risk factor attribution

**Figure 3:**
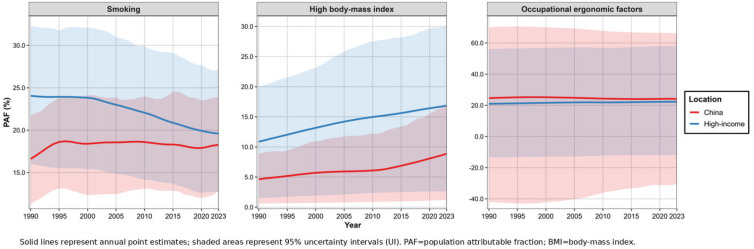
Population attributable fraction (PAF, %) of low back pain YLDs attributable to smoking, high body-mass index, and occupational ergonomic factors among adults aged 20–54 years in China and the GBD aggregate 'High-income countries', 1990–2023 (GBD 2023 comparative risk assessment

**Figure 4:**
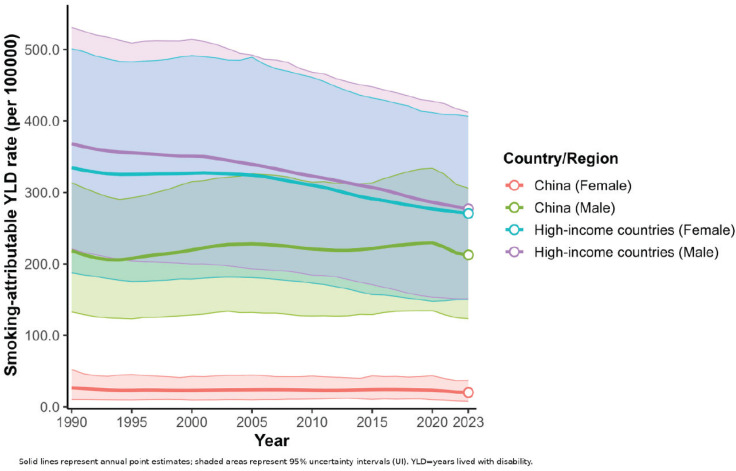
Smoking-attributable age-standardised low back pain YLD rate (per 100000) by sex among adults aged 20–54 years in China and the GBD aggregate 'High-income countries', 1990–2023 (GBD 2023 comparative risk assessment)

Risk-attributable trends in YLD among adults aged 20–54 years in China and high-income countries were decomposed using GBD comparative risk assessment outputs for smoking, high BMI, and occupational ergonomic exposure (Figure 3 and Table 2; and Supplementary file Figure S8). In high-income countries, the smoking-attributable burden declined steadily over 1990–2023 (AAPC= -0.75%; 95% CI: -0.78 – -0.72; p<0.001), accompanied by a reduction in overall PAF from 24.05% to 19.60%. Declines were larger in males (AAPC= -0.86%; 95% CI: -0.88 – -0.83; p<0.001) than females (AAPC= -0.64%; 95% CI: -0.66 – -0.62; p<0.001) (Figure 4), with continued decreases in recent joinpoint segments. By contrast, high BMI-attributable burden increased markedly (AAPC=1.20%; 95% CI: 1.18–1.23; p<0.001), and the overall PAF rose from 10.87% to 16.85%, with similar upward trends in both sexes and evidence of recent deceleration. Occupational ergonomic-attributable burden showed a small net increase (AAPC= 0.04%; 95% CI: 0.02–0.06; p<0.001), with the overall PAF increasing from 20.98% to 22.23% (Table 2). Sex-specific patterns diverged, and recent joinpoint segments indicated an overall shift toward decline (Supplementary file Figure S9).

**Table 2:** Changes in population attributable fractions (PAFs) and risk-attributable YLD rates for low-back pain YLDs among adults aged 20–54 years in China and GBD high-income countries, 1990 and 2023, secondary analysis of GBD 2023 estimates

*Risk factor*	*Sex*	*China*					*High-income countries*				
		*1990 PAF (%) (95% UI)*	*2023 PAF (%) (95% UI)*	*Relative change (%) (95% UI)*	*1990 attributable YLD rate/100k (95% UI)*	*2023 attributable YLD rate/100k (95% UI)*	*1990 PAF (%) (95% UI)*	*2023 PAF (%) (95% UI)*	*Relative change (%) (95% UI)*	*1990 attributable YLD rate/100k (95% UI)*	*2023 attributable YLD rate/100k (95% UI)*
High body-mass index	Both	4.63 (0.58–8.86)	8.87 (1.22–17.11)	91.55 (39.44–180.02)	35.30 (5.23–70.89)	58.28 (8.92–120.88)	10.87 (1.48–19.94)	16.85 (2.64–30.00)	54.98 (31.42–83.47)	159.24 (23.98–320.74)	235.72 (37.43–459.47)
	Male	4.06 (0.51–8.00)	8.65 (1.24–16.59)	113.25 (52.61–204.84)	25.08 (3.71–50.27)	50.65 (7.51–102.69)	11.36 (1.59–20.73)	17.48 (2.76–30.59)	53.95 (28.43–81.26)	140.99 (21.64–276.32)	206.96 (32.99–409.98)
	Female	5.04 (0.64–9.40)	9.05 (1.21–17.41)	79.44 (22.84–161.56)	46.31 (6.84–93.26)	66.48 (10.26–142.22)	10.51 (1.39–19.40)	16.37 (2.56–29.56)	55.79 (32.64–86.05)	177.62 (26.34–362.01)	264.88 (41.93–512.53)
Smoking	Both	16.61 (11.33–21.74)	18.27 (12.85–23.88)	10.01 (-7.13–26.04)	126.19 (75.91–185.51)	119.85 (67.61–173.61)	24.05 (16.04–32.23)	19.60 (12.70–27.14)	-18.50 (-26.56 – -10.48)	351.54 (204.51–520.19)	273.92 (151.13–413.67)
	Male	35.47 (25.27–44.57)	36.37 (26.22–47.47)	2.53 (-8.39–12.44)	218.53 (132.79–313.63)	212.61 (123.40–306.06)	29.72 (20.50–39.05)	23.45 (14.72–31.96)	-21.09 (-29.84 – -13.25)	368.21 (221.27–530.76)	277.17 (151.16–412.45)
	Female	2.94 (1.33–5.91)	2.74 (1.31–4.84)	-6.56 (-53.32–89.75)	26.72 (10.18–52.22)	20.10 (7.85–37.22)	19.86 (12.82–27.19)	16.75 (10.82–23.67)	-15.67 (-25.21 – -5.71)	334.75 (187.63–501.16)	270.62 (150.25–406.56)
Occupational ergonomic factors	Both	24.63 (-41.96–70.05)	24.00 (-30.37–65.92)	-2.53 (-69.84–37.39)	188.41 (-297.77–567.27)	157.66 (-187.37–469.86)	20.98 (-13.41–56.08)	22.23 (-11.85–57.78)	5.98 (-63.87–63.60)	306.98 (-194.89–830.12)	310.33 (-174.97–796.09)
	Male	25.80 (-45.26–72.09)	24.43 (-31.44–67.06)	-5.32 (-52.69–35.95)	160.17 (-269.43–479.90)	143.19 (-173.38–417.50)	25.14 (-20.62–64.68)	24.82 (-16.29–63.51)	-1.26 (-34.46–40.10)	312.06 (-245.38–832.31)	293.30 (-203.40–765.60)
	Female	23.78 (-38.65–68.55)	23.64 (-28.88–65.23)	-0.58 (-77.84–39.70)	218.83 (-337.53–663.93)	173.21 (-200.57–523.32)	17.91 (-8.35–49.71)	20.32 (-9.37–53.13)	13.46 (-125.47–74.68)	301.87 (-150.19–830.92)	327.60 (-139.28–839.38)

PAFs (%) and risk-attributable YLD rates (per 100000 population) are from the GBD 2023 comparative risk assessment and are shown with 95% UI. Relative change (%) = [(2023-1990)/1990]×100. For IHME comparative risk assessment outputs, 95% uncertainty intervals (UI) are derived from model draws and may cross 0; therefore, some risk-attributable YLD rate lower bounds can be negative. These negative values indicate statistical uncertainty around the counterfactual attribution and should be interpreted as compatible with 0 attributable burden, not as a protective effect. PAF: population attributable fraction. YLD: years lived with disability. UI: uncertainty interval. BMI: body-mass index.

In China, trends differed across risk factors. Smoking-attributable burden showed a slight overall decline (AAPC= -0.17%; 95% CI: -0.25 – -0.09; p<0.001), despite an increase in overall PAF from 16.61% to 18.27%. The long-term decline was concentrated among females (AAPC= -0.90%; 95% CI: -1.13 – -0.67; p<0.001), whereas changes in males were comparatively modest (AAPC= -0.10%; 95% CI: -0.16 – -0.03; p<0.05) (Figure 4); however, both sexes exhibited accelerated declines during 2020–2023. High BMI-attributable burden increased significantly (AAPC=1.52%; 95% CI: 1.33–1.70; p<0.001), with the overall PAF rising from 4.63% to 8.87% (Table 2). The increase was faster in males (AAPC=2.15%; 95% CI: 2.02–2.29; p<0.001) than females (AAPC=1.08%; 95% CI: 0.88–1.27; p<0.001), and recent segments suggested a slowdown around 2020–2023. Occupational ergonomic-attributable burden declined over 1990–2023 (AAPC= -0.53%; 95% CI: -0.64 – -0.42; p<0.001), while the overall PAF remained high with only a modest decrease from 24.63% to 24.00% (Table 2), and declines accelerated during 2020–2023 in both sexes (Supplementary file Figure S10).

### Heterogeneity within high-income subregions

To assess whether the high-income aggregate masks heterogeneity relevant to interpreting China versus high-income comparisons, we examined the five GBD-defined high-income subregions (Supplementary file Table S2). In 2023, working-age YLD rates ranged from 1265.22 per 100000 (95% UI: 868.23–1684.30) in Western Europe to 1632.84 (95% UI: 1104.76–2191.04) in Australasia, with intermediate levels in High-income North America (1497.68; 95% UI: 1039.48–1955.99) and High-income Asia Pacific (1493.28; 95% UI: 1020.58–2000.30); Southern Latin America was 1328.03 (95% UI: 916.99–1763.20).

Long-term trends differed across subregions. Over 1990–2023, the AAPC in working-age YLD rates was -0.22% (95% CI: -0.27 – -0.18; p<0.001) in Australasia, -0.21% (95% CI: -0.24 – -0.18; p<0.001) in High-income North America, -0.13% (95% CI: -0.20 – -0.06; p<0.001) in High-income Asia Pacific, and -0.10% (95% CI: -0.11 – -0.08; p<0.001) in Western Europe. Southern Latin America showed no statistically significant long-term change (AAPC= -0.0230%, 95% CI: -0.0701–0.0241; p>0.05) (Supplementary file Table S2). Smoking PAF declined across all five subregions (Supplementary file Table S3). In 1990, subregional smoking PAF ranged from 20.27% (95% UI: 13.46–27.71) in High-income Asia Pacific to 26.09% (95% UI: 17.70–34.76) in Western Europe; by 2023 it ranged from 16.97% (95% UI: 10.05–25.13) in Australasia to 22.51% (95% UI: 15.30–30.95) in Western Europe. The largest relative decline was observed in High-income North America (-25.78; 95% UI: -42.82 – -6.39), whereas declines were smaller and less certain in High-income Asia Pacific (-13.49; 95% UI: -30.02–6.09) and Southern Latin America (-15.11; 95% UI: -39.85–21.68). High BMI and occupational ergonomic PAFs across subregions are reported as contextual comparators in Supplementary file Table S3.

## DISCUSSION

Low-back pain (LBP) remains a leading cause of non-fatal health loss and continues to generate substantial healthcare use and productivity loss during the working years^2,13^. Using GBD 2023 estimates, temporal patterns in China and the GBD aggregate of high-income countries from 1990 to 2023 were examined with a focus on smoking-attributable disability among adults aged 20–54 years, interpreted alongside two co-existing drivers – high BMI and occupational ergonomic exposures. Across this period, working-age age-standardized incidence and YLD rates declined in both settings, with a steeper long-term reduction in China. Smoking-attributable YLD rates also declined in both settings, yet the proportional contribution of smoking diverged: smoking PAF decreased in high-income countries but increased slightly in China. Sex- and age-stratified results, the all-age context, and subregional benchmarking further indicate that the risk structure underlying disability has not converged.

The contrast between attributable rates and PAF is particularly instructive. In high-income countries, a falling smoking-attributable rate together with a falling PAF is compatible with long-running tobacco control and cohort shifts that reduce exposure broadly enough for smoking’s share of disability to shrink. In China, smoking-attributable age-standardized YLD rates decreased while smoking PAF rose slightly. Within the comparative risk framework, this combination is plausible when the overall LBP baseline improves faster than the smoking-attributable component and when smoking exposure remains concentrated in high-impact groups – most notably working-age men – so that proportional contribution does not fall in parallel.

Biological plausibility supports discussing smoking in relation to musculoskeletal disability without reducing LBP to a single cause. Nicotine and other tobacco constituents can promote vasoconstriction and impair microcirculatory perfusion, potentially reducing oxygen and nutrient delivery to disc and paraspinal tissues^17^. In addition, impaired tissue repair and altered immune responses provide a plausible link to slower recovery and longer symptom persistence after back-pain episodes^8^. These mechanisms support the relevance of tobacco control for disability prevention while recognizing that the present analysis is ecological and does not quantify individual-level causal effects.

The non-tobacco drivers help explain why overall burden can improve while prevention priorities shift. High BMI PAF rose in both settings, consistent with the broader expansion of metabolic risk in working-age adults^18^. Higher BMI increases spinal mechanical loading and is associated with adverse body composition and chronic low-grade inflammation, all of which can plausibly contribute to persistent pain and functional limitation^19^. Occupational ergonomic attribution remained substantial, especially in China where ergonomic PAF stayed high despite declining attributable rates, consistent with ongoing biomechanical exposures in parts of the workforce even as average intensity and/or disability duration declines^20^. Sex-stratified attribution patterns suggest that these risks do not evolve uniformly: where BMI attribution rises faster in males (as observed in our China results), smoking and metabolic risk can accumulate in the same subgroups, whereas ergonomic and caregiving-related exposures may remain more salient for women even when smoking exposure is low^21,22^.

Changes in contemporary work organization provide a coherent background for these patterns. Digitalization, remote or hybrid work, and the diffusion of AI-enabled tools have expanded screen-based tasks and prolonged sitting in many occupations, often with reduced posture variation and fewer movement breaks. At the same time, labor markets remain bifurcated – sedentary knowledge work has grown, while physically demanding roles persist in logistics, warehousing, delivery, and care work where lifting, awkward postures, and time pressure remain common^23^. Automation can reduce certain manual loads, yet algorithmic scheduling and monitoring may also intensify pace and compress recovery time. Although these shifts are not separately quantified in the current risk constructs, they provide context for why metabolic and work-related exposures can remain prominent even as age-standardized rates decline.

Beyond attribution, the burden trajectories suggest different stages of transition between settings. High-income countries showed modest long-term declines in working-age rates with recent acceleration in joinpoint-defined segments, whereas China exhibited larger long-term reductions and a marked post-2020 inflection. This turning point was observed across incidence, prevalence, and YLD metrics and in both men and women^24^. Because this segment overlaps the COVID-19 period, it is prudent to interpret it primarily as a period effect: changes in care-seeking, reporting, and case capture, health-service utilization, mobility and physical activity, and work arrangements (including telework for some groups) could influence modeled estimates without implying a single causal pathway^25^. Continued surveillance is needed to determine whether post-2020 slopes persist as service use and labor patterns stabilize ^26,27^.

Interpretation should also consider differences in input data and model dependence across settings. GBD estimates synthesize heterogeneous sources, and data density and representativeness may differ between China and high-income locations^6^. The contribution of covariates and borrowing can therefore vary by period and metric. This may influence uncertainty and the stability of trend and attribution estimates, particularly for comparative risk assessment outputs^28^.

Sex-stratified trends refine interpretation and highlight leverage points. In high-income countries, larger smoking-attributable declines among males align with historically higher exposure and greater scope for reduction. In China, comparatively modest long-term change among males is consistent with the persistent sex gap in smoking exposure, while female declines suggest a different exposure distribution and competing risk structure^22^. For non-tobacco drivers, differences in occupational composition and task allocation plausibly contribute to sex gaps in ergonomic attribution, while sex differences in metabolic transition may contribute to divergent BMI-attributable trajectories^21,29^.

Age patterns and the all-age context clarify why working-age disability deserves emphasis while also underscoring service-planning pressures. Adults aged 20–54 years accounted for roughly 42–43% of all-age LBP YLD in 2023 in both settings, concentrating disability during peak working years. High-income countries showed a stable working-age gradient, whereas China’s profile was more stepwise, consistent with cohort- and period-specific dynamics during rapid transition^30^. At the all-age level, age-standardized rates declined while absolute YLD counts increased in both settings, reflecting population growth and ageing^31^.

Subregional benchmarking further cautions against treating the high-income aggregate as a single epidemiologic trajectory. Working-age YLD levels and long-term trends varied across high-income subregions, and smoking PAF declined in all five subregions, indicating a consistent direction of tobacco transition across high-income settings. In contrast, broadly rising BMI PAF across subregions points to a shared metabolic shift that may increasingly constrain further reductions in disability unless addressed alongside sustained tobacco control and workplace risk mitigation^32^.

Overall, the findings support an integrated prevention frame at the population level. The observed attribution patterns may help prioritize cessation support within broader strategies that also address BMI-related risk and work-related exposures, while recognizing that model-based ecological analyses cannot demonstrate the effectiveness of specific interventions^30,33^. Clinically, integrating brief cessation support and weight-management counselling into musculoskeletal care pathways, alongside workplace-oriented prevention addressing both biomechanical hazards and sedentary work configurations, may contribute to reducing disability during the working years^34^.

### Limitations

This study has limitations inherent to GBD analyses. First, estimates are modeled and depend on data availability and modeling assumptions; uncertainty may be substantial, particularly for risk-attribution estimates. Second, GBD quantifies non-specific LBP and does not capture clinical subtypes or local diagnostic practices, which may affect comparability across settings. Third, the ecological, model-based design does not allow individual-level causal inference, especially regarding smoking and LBP burden. Fourth, risk attribution was limited to GBD-modeled risks and does not include all relevant determinants (e.g. mental health, physical activity, and psychosocial factors). Finally, joinpoint trend estimates were based on annual point estimates and did not propagate GBD 95% uncertainty intervals into APC/AAPC; subregional analyses were restricted to avoid over-stratification.

## CONCLUSIONS

From 1990 to 2023, age-standardized LBP incidence and YLD rates among adults aged 20–54 years declined in both China and high-income countries, with larger reductions in China. Smoking-attributable YLD rates declined in both settings, but smoking’s proportional contribution decreased in high-income countries and rose slightly in China, indicating divergent risk composition. Over the same period, high BMI attribution increased and occupational ergonomic exposure remained substantial, particularly in China. These findings may help prioritize tobacco control and cessation support within broader strategies that also address BMI-related and work-related risks for working-age disability.

## Supplementary Material



## Data Availability

All data used in this study are publicly available from the IHME Global Burden of Disease (GBD) Results Tool (GBD 2023). The analytic code and processed datasets used to generate the figures and tables are available from the authors upon reasonable request.
