# Supplementary material for: Smoking-attributable low-back pain disability in China versus high income countries among adults aged 20–54 years, 1990–2023: A secondary dataset analysis of GBD 2023
Source: Tob Induc Dis. 2026 Jun 12;24:10.18332/tid/219036. doi: 10.18332/tid/219036 (PMC13261638; doi:10.18332/tid/219036)
Supplement: Supplementary file 1 [file TID-24-91-s1.pdf]

Supplement

Tables

Table S1. Low back pain burden in the all-age population in China and GBD high-income countries, 1990–2023 (secondary analysis of GBD 2023 estimates).

Location	Measure	1990 Cases	1990 Rate/100k (95% UI)	2023 Cases	2023 Rate/100k (95% UI)	Cases Change, % (95% UI)	AAPC (95% CI)
**China**	Incidence	29,989,124(26,335,876-33,418,905)	2,859.73 (2,532.11-3,187.09)	41,383,565(36,417,179-46,520,393)	2,164.80 (1,926.65-2,383.57)	38.00 (30.88 to 45.57)	-0.84* (-0.89 to -0.79)
	Prevalence	68,636,309(59,816,847-77,457,397)	6,636.60 (5,778.62-7,462.02)	95,323,956(83,625,063-107,778,790)	4,929.78 (4,304.39-5,494.10)	38.88 (32.28 to 46.82)	-0.90* (-0.95 to -0.84)
	YLDs	7,732,428(5,438,615-10,477,031)	740.83 (525.01-1,010.60)	10,635,869(7,520,420-14,715,745)	551.92 (388.90-750.82)	37.55 (30.39 to 45.05)	-0.89* (-0.95 to -0.83)
**High-income**	Incidence	45,973,775(41,204,151-50,904,463)	4,448.43 (3,977.10-4,941.23)	60,763,100(54,540,314-66,829,456)	4,166.31 (3,749.45-4,563.83)	32.17 (28.73 to 36.16)	-0.19* (-0.21 to -0.17)
	Prevalence	110,383,257(98,897,173-121,775,256)	10,566.66 (9,478.84-11,743.26)	147,119,809(131,865,369-160,711,705)	9,844.18 (8,911.50-10,790.61)	33.28 (29.41 to 37.52)	-0.21* (-0.24 to -0.18)
	YLDs	12,311,458(8,667,972-16,616,283)	1,181.87 (832.15-1,591.08)	16,167,801(11,495,490-21,713,802)	1,095.83 (775.15-1,467.99)	31.32 (27.54 to 35.53)	-0.22* (-0.25 to -0.20)

Note: Rates are age-standardised per 100,000 population with 95% UI. Cases change (%) = (2023 − 1990)/1990 × 100. AAPC (1990–2023) was estimated from Joinpoint regression applied to annual point estimates and is reported with 95% CI. *Statistically significant (P<0.05). AAPC, average annual percent change; CI, confidence interval; UI, uncertainty interval; YLD, years lived with disability.

Table S2. Working-age (20–54 years) low back pain YLDs in five GBD high-income subregions, 1990 and 2023, and AAPC (1990–2023) (secondary analysis of GBD 2023 estimates).

High-income subregion	1990 YLDs, number (95% UI)	1990 YLD rate per 100,000 (95% UI)	2023 YLDs, number (95% UI)	2023 YLD rate per 100,000 (95% UI)	AAPC, % (95% CI)
Australasia	177,650 (119,368–243,151)	1,762.77 (1,184.46–2,412.72)	244,782 (165,617–328,463)	1,632.84 (1,104.76–2,191.04)	−0.22* (−0.27 to −0.18)
High-income North America	2,279,623 (1,589,140–3,044,601)	1,606.64 (1,120.00–2,145.79)	2,542,340 (1,764,528–3,320,327)	1,497.68 (1,039.48–1,955.99)	−0.21* (−0.24 to −0.18)
High-income Asia Pacific	1,359,929 (940,332–1,832,191)	1,562.43 (1,080.35–2,105.01)	1,214,996 (830,384–1,627,527)	1,493.28 (1,020.58–2,000.30)	−0.13* (−0.20 to −0.06)
Western Europe	2,471,519 (1,713,452–3,330,192)	1,305.47 (905.05–1,759.02)	2,505,659 (1,719,454–3,335,621)	1,265.22 (868.23–1,684.30)	−0.10* (−0.11 to −0.08)
Southern Latin America	300,871 (202,136–407,431)	1,346.73 (904.78–1,823.70)	462,162 (319,119–613,607)	1,328.03 (916.99–1,763.20)	−0.02 (−0.07 to 0.02)

Note: YLD counts and YLD rates (per 100,000 population) are for adults aged 20–54 years (both sexes) with 95% UI. AAPC for YLD rates over 1990–2023 was estimated using Joinpoint regression and is reported with 95% CI. *Statistically significant (P<0.05). AAPC, average annual percent change; CI, confidence interval; UI, uncertainty interval; YLD, years lived with disability.

Table S3. Population attributable fractions (PAFs) of low back pain YLDs by risk factor across GBD high-income subregions, 1990 and 2023 (secondary analysis of GBD 2023 estimates).

Subregion	Risk factor	PAF, % (95% UI)		Change	
		1990	2023	Absolute, % (pp)	Relative, % (95% UI)
Australasia	Smoking	21.55 (13.66-29.97)	16.97 (10.05-25.13)	-4.58	-21.27 (-39.89 to 3.26)
	High BMI	11.53 (1.60-21.49)	18.74 (3.11-34.09)	7.20	62.46 (19.77 to 115.64)
	Occupational ergonomic	21.17 (-12.84-57.02)	22.25 (-11.66-55.92)	1.08	5.09 (-99.76 to 91.40)
High-income North America	Smoking	24.72 (16.36-33.21)	18.35 (11.05-26.54)	-6.37	-25.78 (-42.82 to -6.39)
	High BMI	14.72 (2.19-26.97)	22.07 (3.59-38.95)	7.35	49.94 (38.91 to 67.08)
	Occupational ergonomic	22.05 (-12.35-57.28)	22.53 (-12.12-56.87)	0.48	2.18 (-69.52 to 42.14)
High-income Asia Pacific	Smoking	20.27 (13.46-27.71)	17.54 (11.08-25.50)	-2.73	-13.49 (-30.02 to 6.09)
	High BMI	4.58 (0.58-8.49)	7.89 (1.29-15.07)	3.31	72.19 (22.30 to 140.42)
	Occupational ergonomic	21.01 (-16.79-57.82)	21.85 (-12.68-57.91)	0.84	3.99 (-51.76 to 56.30)
Western Europe	Smoking	26.09 (17.70-34.76)	22.51 (15.30-30.95)	-3.59	-13.74 (-22.31 to -6.34)
	High BMI	10.48 (1.34-20.09)	14.97 (2.19-26.91)	4.49	42.90 (10.15 to 87.81)
	Occupational ergonomic	20.32 (-11.80-54.30)	22.23 (-11.70-57.00)	1.91	9.40 (-90.46 to 66.57)
Southern Latin America	Smoking	20.78 (11.67-29.90)	17.64 (10.30-25.72)	-3.14	-15.11 (-39.85 to 21.68)

	High BMI	12.90 (1.62-25.00)	20.65 (3.50-36.78)	7.75	60.06 (15.61 to 132.21)
	Occupational ergonomic	18.07 (-16.00-53.26)	21.63 (-18.16-59.48)	3.57	19.74 (-30.73 to 48.36)

Note: PAFs (%) are from the GBD 2023 comparative risk assessment for adults aged 20–54 years (both sexes) and are shown with 95% UI. Absolute change (percentage points, pp) = PAF[2023] – PAF[1990]. Relative change (%) = (PAF[2023] – PAF[1990])/PAF[1990] × 100. PAF, population attributable fraction; UI, uncertainty interval; YLD, years lived with disability; BMI, body-mass index.

Figures

Figure S1. Age-standardised low back pain incidence(A) and(B) prevalence rate (per 100,000) among adults aged 20–54 years in China and the GBD aggregate "High-income countries", 1990–2023 (GBD 2023 estimates).

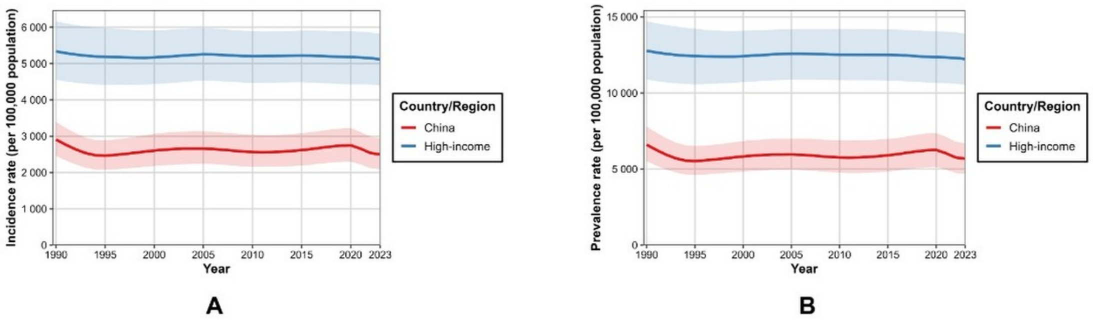

Solid lines represent annual point estimates; shaded areas represent 95% uncertainty intervals (UI).

Figure S2. Joinpoint-derived annual percent change (APC) in age-standardised LBP incidence, prevalence and YLD rates among adults aged 20–54 years in China and high-income countries, 1990–2023. (A) APC in incidence rates for the high-income country population. (B) APC in prevalence rates for the high-income country population. (C) APC in years lived with disability (YLDs) rates for the high-income country population. (D) APC in incidence rates for the Chinese population. (E) APC in prevalence rates for the Chinese population. (F) APC in years lived with disability (YLDs) rates for the Chinese population.

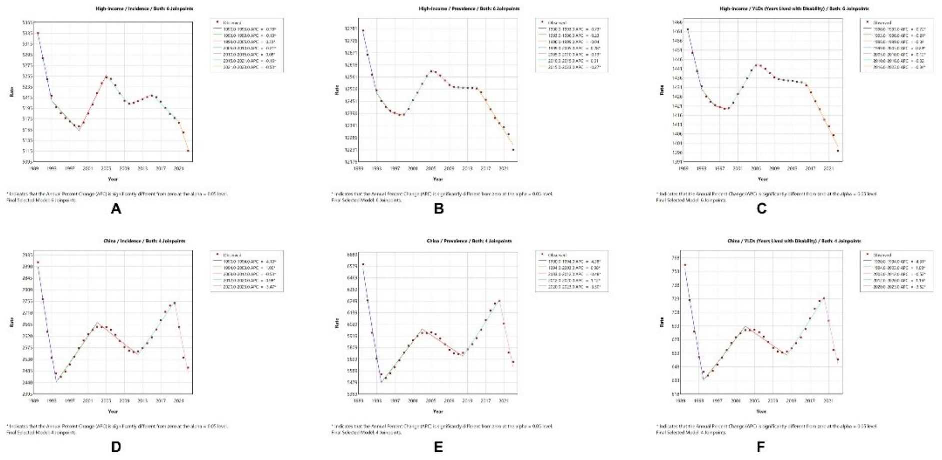

Figure S3. Sex-specific age-standardised low back pain incidence(A), prevalence(B) and YLD(C) rates (per 100,000) among adults aged 20–54 years in China and the GBD aggregate "High-income countries", 1990–2023 (GBD 2023 estimates).

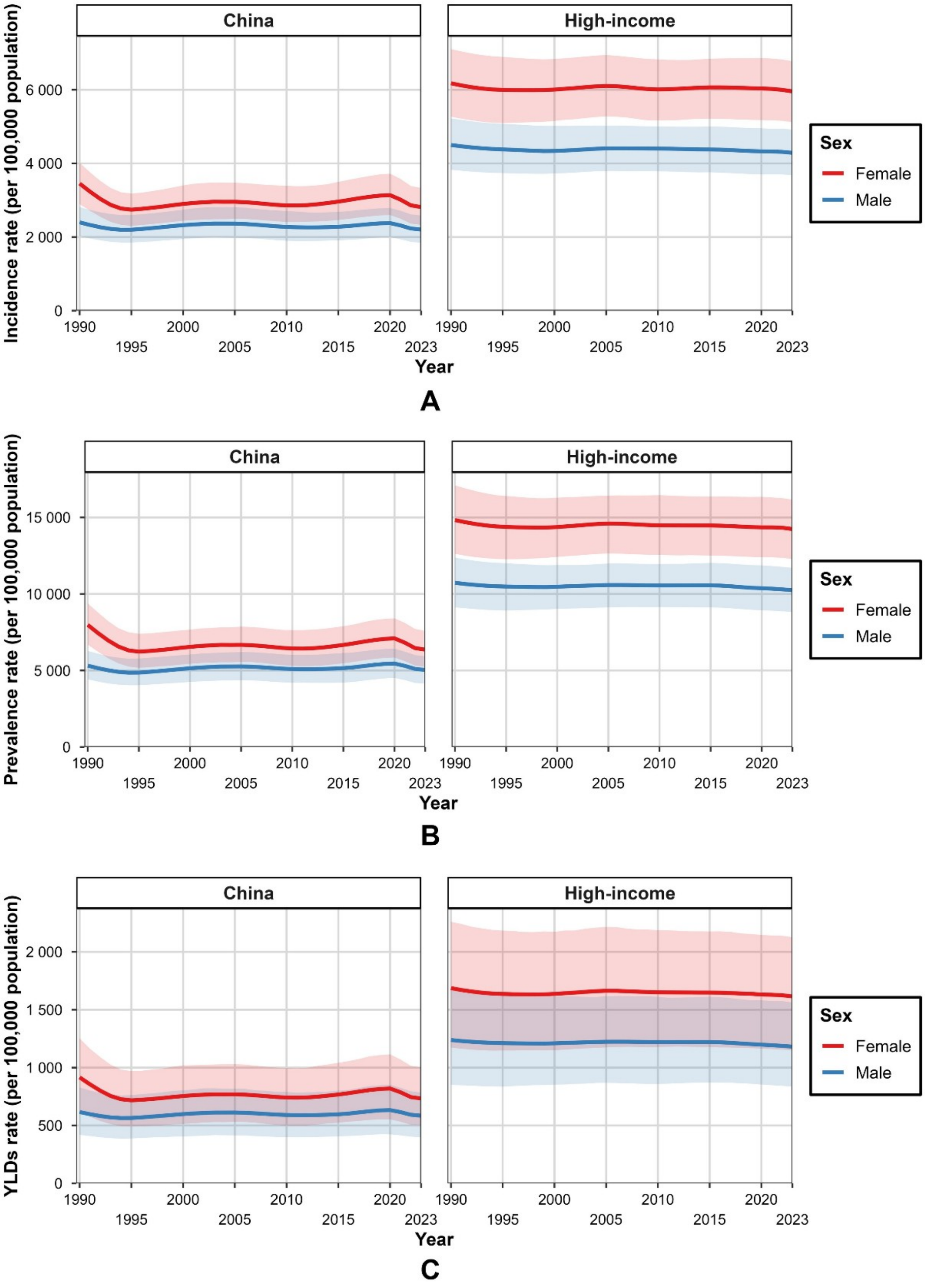

Solid lines represent annual point estimates; shaded areas represent 95% uncertainty intervals (UI). YLD=years lived with disability.

Figure S4. Secondary analysis of publicly available GBD 2023 estimates: Joinpoint-derived annual percent change (APC) in age-standardised low back pain incidence, prevalence, and years lived with disability (YLDs) rates among adults aged 20–54 years in the GBD aggregate “High-income countries”, 1990–2023. (A) APC in incidence rates for females in high-income countries. (B) APC in prevalence rates for females in high-income countries. (C) APC in YLDs rates for females in high-income countries. (D) APC in incidence rates for males in high-income countries. (E) APC in prevalence rates for males in high-income countries. (F) APC in YLDs rates for males in high-income countries.

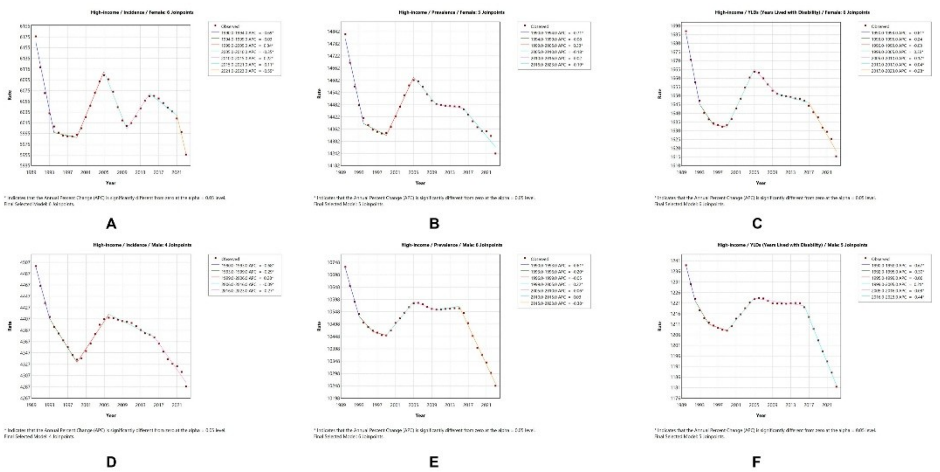

Figure S5. Secondary analysis of publicly available GBD 2023 estimates: Joinpoint-derived annual percent change (APC) in age-standardised low back pain incidence, prevalence, and years lived with disability (YLDs) rates among adults aged 20–54 years in China, 1990–2023. (A) Annual percentage change in incidence rates for females in China. (B) Annual percentage change in prevalence rates for females in China. (C) APC in YLDs rates for females in China. (D) APC in incidence rates for males in China. (E) APC in prevalence rates for males in China. (F) APC in YLDs rates for males in China.

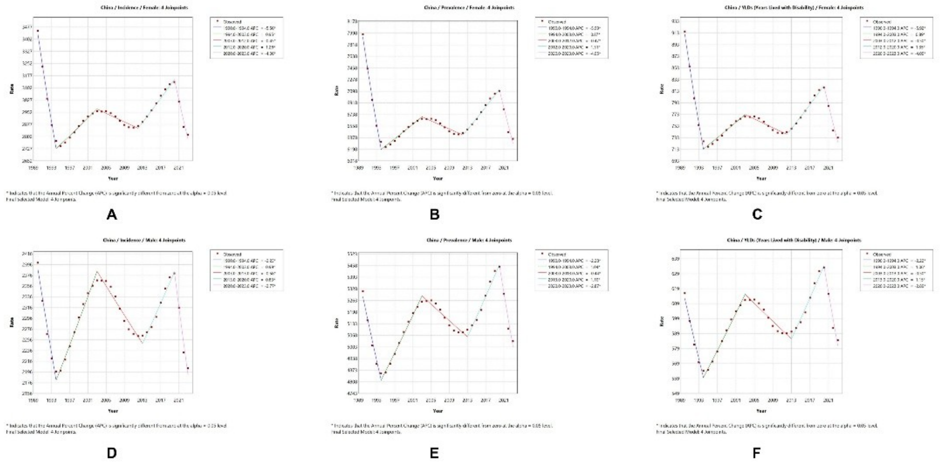

Figure S6. Age-standardised low back pain incidence(A), prevalence(B) and YLD(C) rates (per 100,000) across all ages in China and the GBD aggregate "High-income countries", 1990–2023 (GBD 2023 estimates).

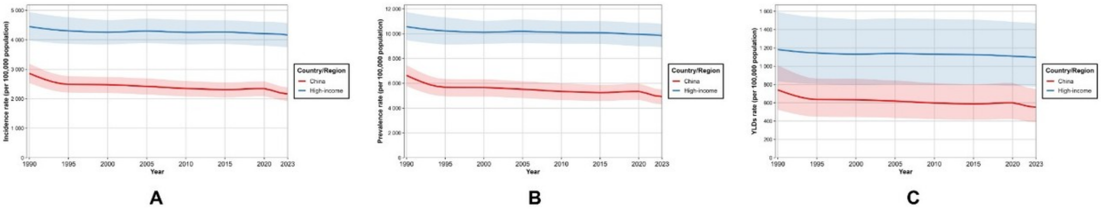

Solid lines represent annual point estimates; shaded areas represent 95% uncertainty intervals (UI). YLD=years lived with disability.

Figure S7. Low back pain YLD rate (per 100,000) by age group in China and the GBD aggregate "High-income countries" (GBD 2023 estimates).

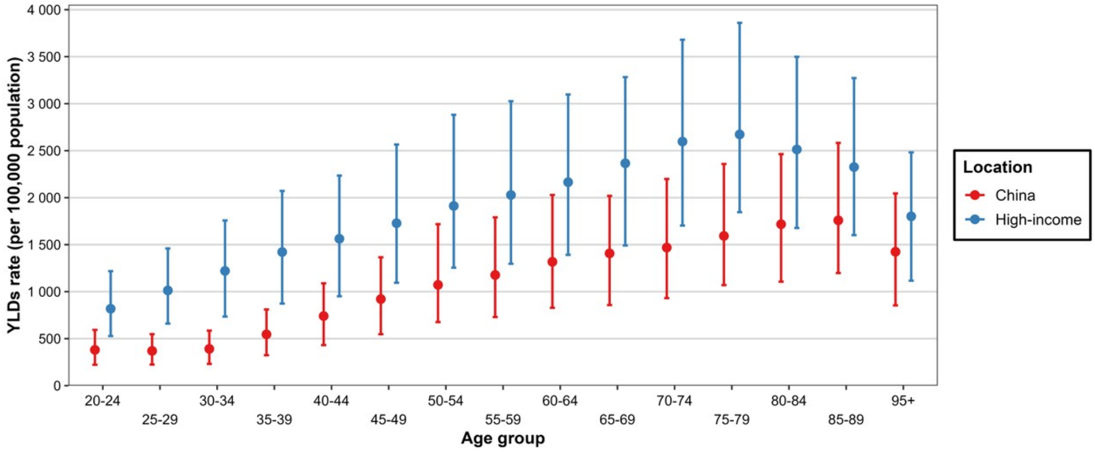

Points represent YLD rates by age group; error bars indicate 95% uncertainty intervals (UI). YLD=years lived with disability.

Figure S8. Secondary analysis of GBD 2023 comparative risk assessment: Joinpoint-derived APC in age-standardised risk-attributable low back pain YLD rates among adults aged 20–54 years in China and the GBD aggregate "High-income countries", 1990–2023. (A) APC for smoking-attributable YLDs rate in high-income countries. (B) APC for high BMI-attributable YLDs rate in high-income countries. (C) APC for occupational ergonomics-attributable YLDs rate in high-income countries. (D) APC for smoking-attributable YLDs rate in China. (E) APC for high BMI-attributable YLDs rate in China. (F) APC for occupational ergonomics-attributable YLDs rate in China.

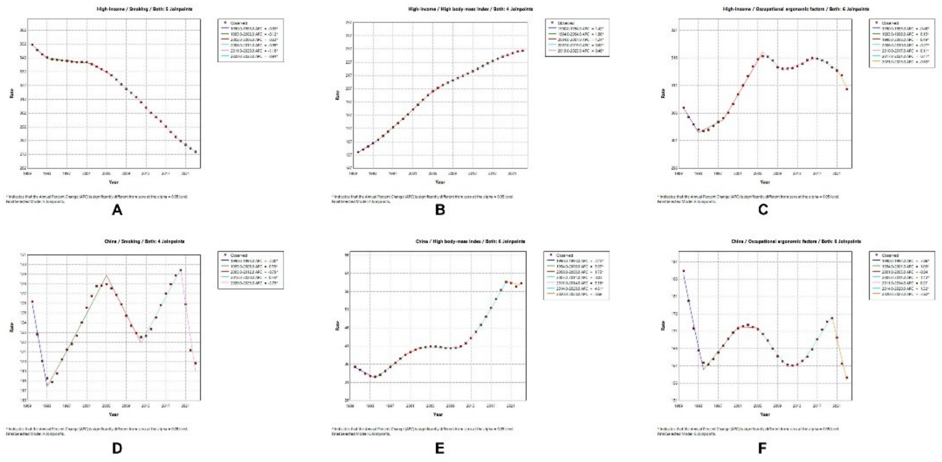

Figure S9. Secondary analysis of GBD 2023 comparative risk assessment: Joinpoint-derived APC in age-standardised risk-attributable low back pain YLD rates by sex among adults aged 20–54 years in the GBD aggregate "High-income countries", 1990–2023. (A) APC for smoking-attributable YLDs rate in males in high-income countries. (B) APC for smoking-attributable YLDs rate in females in high-income countries. (C) APC for high BMI-attributable YLDs rate in males in high-income countries. (D) APC for high BMI-attributable YLDs rate in females in high-income countries. (E) APC for occupational ergonomics-attributable YLDs rate in males in high-income countries. (F) APC for occupational ergonomics-attributable YLDs rate in females in high-income countries.

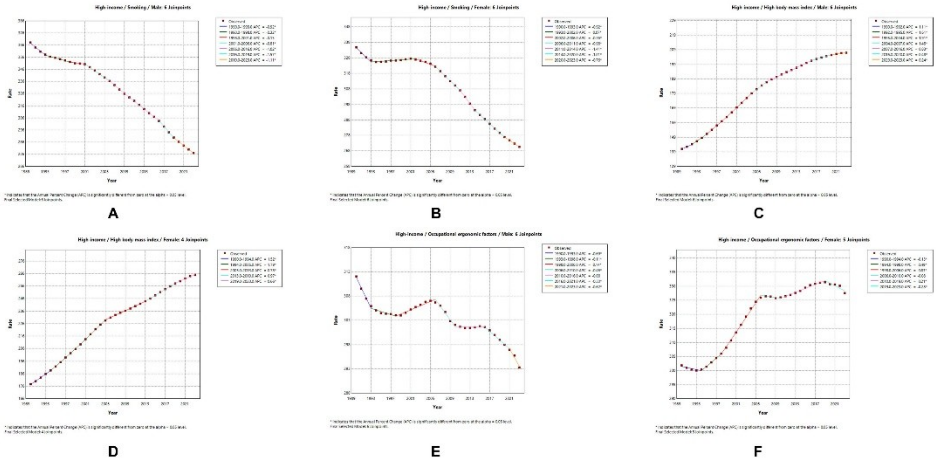

Figure S10. Secondary analysis of GBD 2023 comparative risk assessment: Joinpoint-derived APC in age-standardised risk-attributable low back pain YLD rates by sex among adults aged 20–54 years in China, 1990–2023. (A) APC for smoking-attributable YLDs rate in males in China. (B) APC for smoking-attributable YLDs rate in females in China. (C) APC for high BMI-attributable YLDs rate in males in China. (D) APC for high BMI-attributable YLDs rate in females in China. (E) APC for occupational ergonomics-attributable YLDs rate in males in China. (F) APC for occupational ergonomics-attributable YLDs rate in females in China.

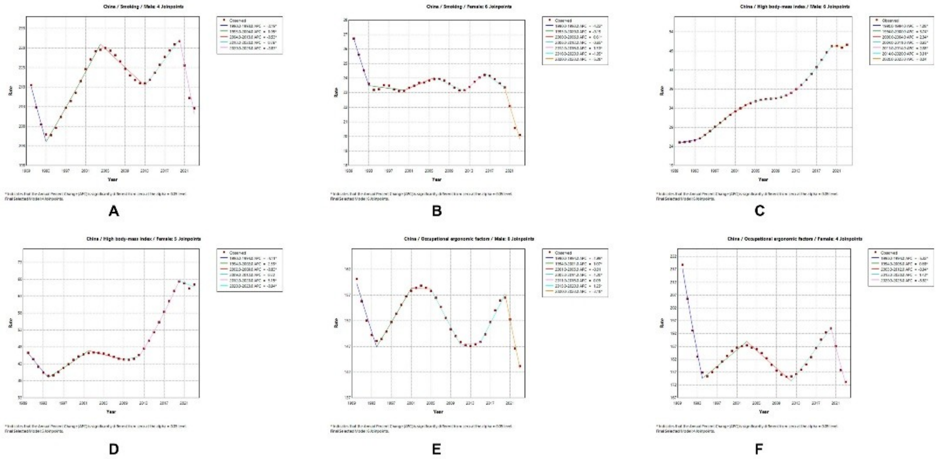

© 2026 Zhu Y. et al.
